
JOURNAL INFORMATION
==============================
NLM Title Abbreviation: ScientificWorldJournal
Journal ID: ScientificWorldJournal
NLM Journal ID: 101131163
Journal ID: TSWJ

The Scientific World Journal

ISSN: 2356-6140
EISSN: 1537-744X
Publisher: Wiley

ARTICLE INFORMATION
==============================
PMCID: PMC5901198
PMID: 29861693
DOI: 10.1100/tsw.2007.227
Article ID: 968275
Article version: 1
Subjects: Editorial

Sleep, Circadian Rhythm, and Drug Abuse

Gordon Hal W. *
The Neuroscience Center, Room 3168, MSC 9593, 6001 Executive Boulevard, Bethesda, MD 20892-9593, USA
*Harold W. Gordon: hgordon1@nida.nih.govAcademic Editors: D. Shurtleff and S. Ferre

Publication date: 2007
Electronic publication date: 2007 Nov 2
Volume: 7
First page: 191
Last page: 193
Copyright: Copyright © 2007 Hal W. Gordon.
Copyright year: 2007
License: This is an open access article distributed under the Creative Commons Attribution License, which permits unrestricted use, distribution, and reproduction in any medium, provided the original work is properly cited.
License URL: https://creativecommons.org/licenses/by/3.0/

==============================
Editorial
NIDA Special Issue on Frontiers in Addiction Research
*TheScientificWorldJOURNAL* (2007) 7(S2), 191–193
ISSN 1537-744X; DOI 10.1100/tsw.2007.227

TheScientificWorldJOURNAL
www.thescientificworld.com

### Sleep, Circadian Rhythm, and Drug Abuse

Harold W. Gordon

*The Neuroscience Center, Room 3168, MSC 9593, 6001 Executive Boulevard, Bethesda, MD 20892-9593, USA*

**E-mail**: hgordon1@nida.nih.gov

*Published November 2, 2007*

---

*Question*: When is research on sleep and circadian rhythms not research on sleep and circadian rhythms?

*Answer*: When it is research on any one of a spectrum of diseases and disease conditions that co-occur with disordered sleep and disrupted rhythms.

Instead, the biology and neurobiology underlying the sleep disturbance is inextricably interwoven with the pathophysiology of the disease process itself. So intertwined are these co-occurring conditions that it is often impossible to determine cause and effect. Accordingly, it is difficult to know which condition to treat, much less to know how treatment of one affects the other. This is why the study of sleep should really be a study of the disease, with sleep as one of the biological markers. Conversely, it is equally insufficient to study the underlying pathology of a disease without including consideration of the associated effects on the sleep architecture or on natural (e.g., circadian) rhythms.

Certainly, the study of the physiological and neurological mechanisms that govern sleep and natural rhythms is of significance. One cannot understand insomnia and other sleep disturbances or any of the phase shifts associated with natural ultradian (e.g., 90-min sleep stages), circadian (daily), monthly, or seasonal cycles without understanding the pacemaker mechanisms involved. Indeed, the sleep cycle is crucially related to the health and well-being throughout the animal kingdom. Humans can survive without food longer than they can survive without sleep. Sleep, like food, sustains life; disordered sleep, like lack of nutrition, has adverse consequences for cognitive, mental, and physical health. Nevertheless, millions of people suffer from sleep disorders; many, if not most, of which are undiagnosed. Yet, in spite of its prominence in daily life and the role it plays in maintaining homeostasis, research on sleep has a short history of only about one half century.

In spite of its importance, or perhaps because of it, it is not sufficient to study sleep in isolation, particularly as it relates to health. According to a recent report by the Institute of Medicine[1], significant gaps exist in the study of sleep disorders and their associated diseases. It is estimated that about 90 distinct sleep disorders can be recognized that are associated with such diverse diseases as hypertension, diabetes, obesity, depression, and drug abuse. It is important to realize that the connection is not trivial as these commonly heard statements illustrate: “Lack of sleep puts strain on my heart.” “I can’t sleep so I’ll get something to eat.” “I can sleep better if I have a shot or two of whiskey before I go to bed.” “I’m not depressed, I’m just suffering from lack of sleep.”

A clear example of shortsighted consideration of sleep and circadian mechanisms has occurred in the more than 3-decade study of drug abuse and addiction. Sleep disturbance has long been observed by clinicians as a chronic symptom of drug-taking and withdrawal. In the case of abstinence, the effects outlast the more common symptoms of withdrawal and often precipitate relapse. Nevertheless, it was only

©2007 with author.
Published by TheScientificWorld; www.thescientificworld.com until recently that research focused specifically on circadian rhythm or sleep architecture as integrally related to the disease of addiction itself. In the articles that follow, the first fruits of this research are reported. The results demonstrate that circadian cycles of gene expression influence, or are influenced by, drugs of abuse or their neurological pathways. Similarly, it is found that drugs of abuse modify neurosystems that directly affect the sleep architecture and, therefore, sleep itself. There are suggestions that the consequences impact not only on a patient's propensity for relapse, but also on which treatment to select such that a medication for drug abuse does not exacerbate the sleep disturbance.

The cyclicity of gene expression that makes up the biological clock and its role in the regulation of drug sensitivity, sensitization, and reward is discussed for the animal model in the article by McClung[2]; entrainment of circadian rhythms by drugs of abuse is reported by Kosobud[3]. Not only do drugs of abuse shift this rhythm, but also sensitivity to drugs varies according to a diurnal cycle. These influences may well have consequences both in terms of development of addiction, but also in the effectiveness of treatment. While it has long been known that light is the classic *zeitgeber* (time-giver) that sets biological clocks, Kosobud describes studies in which several drugs of abuse, including opiates, nicotine, stimulants, and alcohol, also entrain a rhythm in rats independent of light[3]. It remains to be determined whether this entrainment in animals implies that there are also compensatory mechanisms which, in humans, might induce enhanced drug-seeking behaviors that contribute to addiction.

Psychostimulants directly affect sleep as pointed out in the article by McCann and Ricuarte and documented in the articles by Valladares and Irwin, and by Morgan and Malison[4,5,6]. McCann and Ricuarte raise the question of whether MDMA, a popular drug of abuse, affects circadian and sleep cycles by its neurotoxic effects on serotonin. Both preclinical and clinical studies are reviewed with the conclusion that MDMA undoubtedly plays a role, but the mechanism remains to be determined. The article by Trksak, Renshaw, and Lukas reports first attempts to assess drug effects at the molecular level using magnetic resonance spectroscopy (MRS), which measures cortical bioenergetics[7]. In particular, greater enhancements in nucleoside triphosphates were seen during recovery in both methadone-maintained and cocaine-dependent, sleep-deprived patients compared to controls, suggesting an adverse effect in these drug-dependent subjects on sleep homeostasis that may involve adenosine triphoshate levels.

Few other studies have directly assessed the effect of drugs of abuse on the neuromechanisms of sleep as has MRS imaging. Nevertheless, based on preclinical models and their own results in chronic cocaine users, Morgan and Malison hypothesize a mechanism involving the GABA system. They document deteriorating sleep and deficient behavioral performance following a 2.5-week abstinence while, at the same time, the subjects denied feeling that their quality of sleep and their performance ability had suffered. These contrasting findings suggested a cognitive disconnect that the authors called "occult insomnia." In addition, they noted that women (all of whom were tested in the luteal phase) did not have the same sleep deterioration following abstinence as men. Therefore, it was hypothesized that, in addition to the GABA system, natural hormones (e.g., progesterone) may also play a role. Most importantly, the analyses of modifications in the sleep architecture that led to hypotheses regarding the underlying neural mechanisms also led to hypotheses regarding pharmacological treatment for addiction. This is a clear example where knowledge of the neurobiology underlying sleep informs on the neurobiology underlying drug abuse thereby pointing to an effective means of treatment.

These studies that focus on circadian rhythms or sleep in the animal model or in the drug-dependent patient illustrate the importance of studying both areas simultaneously. Disordered sleep in dependent patients can inform on the neural effects of psychoactive drugs. Conversely, neural mechanisms underlying the effects of drugs can inform on neurobiology of sleep architecture and circadian neurophysiology. Most importantly for the National Institute on Drug Abuse, new research that considers sleep and circadian factors together in a study of addiction opens up a new interdisciplinary field of research. The research reported here is only the beginning. It highlights the fact that scientists who believe they are only doing research into biorhythms are really doing drug abuse research as well, but don't know it.

### REFERENCE

1. Colten, H.R. and Altevogt, B.M., Eds. (2006) *Sleep Disorders and Sleep Deprivation: An Unmet Public Health Problem*. The National Academies Press, Washington, D.C.
2. McClung, C.A. (2007) Circadian Rhythms, the Mesolimbic Dopaminergic Circuit, and Drug Addiction. *TheScientificWorldJOURNAL* **7(S2)** xx-xx.
3. Kosobud, A.E.K., Gillman, A.G., Leffel II, J.K., Pecoraro, N.C., Rebec, G.V., and Timberlake, W. (2007) Drugs of abuse can entrain circadian rhythms. *TheScientificWorldJOURNAL* **7(S2)** xx-xx.
4. McCann, U.D. and Ricaurte, G.A. (2007) Effects of (±) 3,4-Methylenedioxymethamphetamine (MDMA) on Sleep and Circadian Rhythms. *TheScientificWorldJOURNAL* **7(S2)** xx-xx.
5. Valladares, E.M. and Irwin, M.R. (2007) Polysomnographic Sleep Dysregulation in Cocaine Dependence: Mini-Review. *TheScientificWorldJOURNAL* **7(S2)** xx-xx.
6. Morgan, P.T. and Malison, R.T. (2007) Cocaine and sleep: early abstinence. *TheScientificWorldJOURNAL* **7(S2)** xx-xx.
7. Trksak, G.H., Renshaw, P.F., and Lukas, S.E. (2007) Brain Phosphorus Magnetic Resonance Spectroscopy Imaging of Sleep Homeostasis and Restoration in Drug-Dependence. *TheScientificWorldJOURNAL* **7(S2)** xx-xx.

**This article should be cited as follows:**

Gordon, H.W. (2007) Sleep, circadian rhythm, and drug abuse. *TheScientificWorldJOURNAL* **7(S2)**, 191–193. DOI 10.1100/tsw.2007.227.
